# Structure Activity Relationships of *N*-linked and Diglycosylated Glucosamine-Based Antitumor Glycerolipids

**DOI:** 10.3390/molecules181215288

**Published:** 2013-12-10

**Authors:** Makanjuola Ogunsina, Hangyi Pan, Pranati Samadder, Gilbert Arthur, Frank Schweizer

**Affiliations:** 1 Department of Chemistry, University of Manitoba, 144 Dysart Rd, Winnipeg, MB R3T 2N2, Canada; 2 Department of Biochemistry and Medical Genetics, University of Manitoba, 745 Bannatyne Avenue, Winnipeg, MB R3E 0J9, Canada

**Keywords:** antitumor ether lipids, glycolipids, anticancer agents, carbohydrates

## Abstract

1-*O*-Hexadecyl-2-*O*-methyl-3-*O*-(2'-amino-2'-deoxy-β-d-glucopyranosyl)-*sn*-glycerol (**1**) was previously reported to show potent *in vitro* antitumor activity on a range of cancer cell lines derived from breast, pancreas and prostate cancer. This compound was not toxic to mice and was inactive against breast tumor xenografts in mice. This inactivity was attributed to hydrolysis of the glycosidic linkage by glycosidases. Here three *N*-linked (glycosylamide) analogs **2**–**4**, one triazole-linked analog **5** of **1** as well as two diglycosylated analogs **6** and **7** with different stereochemistry at the C2-position of the glycerol moiety were synthesized and their antitumor activity against breast (JIMT-1, BT-474, MDA-MB-231), pancreas (MiaPaCa2) and prostrate (DU145, PC3) cancer cell lines was determined. The diglycosylated analogs 1-*O*-hexadecyl-2(*R*)-, 3-*O*-di-(2'-amino-2'-deoxy-β-d-glucopyranosyl)-*sn*-glycerol (**7**) and the 1:1 diastereomeric mixture of 1-*O*-hexadecyl-2(*R/S*), 3-*O*-di-(2'-amino-2'-deoxy-β-d-glucopyranosyl)-*sn*-glycerol (**6**) showed the most potent cytotoxic activity at CC_50_ values of 17.5 µM against PC3 cell lines. The replacement of the *O*-glycosidic linkage by a glycosylamide or a glycosyltriazole linkage showed little or no activity at highest concentration tested (30 µM), whereas the replacement of the glycerol moiety by triazole resulted in CC_50_ values in the range of 20 to 30 µM. In conclusion, the replacement of the *O*-glycosidic linkage by an *N*-glycosidic linkage or triazole-linkage resulted in about a two to three fold loss in activity, whereas the replacement of the methoxy group on the glycerol backbone by a second glucosamine moiety did not improve the activity. The stereochemistry at the C2-position of the glycero backbone has minimal effect on the anticancer activities of these diglycosylated analogs.

## 1. Introduction

Cancer is a devastating disease with significant mortality and morbidity in both developed and developing countries [1,2]. Acquired and intrinsic resistance to major classes of anticancer agents: antimetabolites, anthracyclines, taxanes and alkylating agents which are mostly pro-apoptotic present a serious challenge to management of cancer [3]. Even targeted antibody based therapies such as trastuzumab (Herceptin) are also affected by the problem of drug resistance [3,4]. So there is need for new anticancer agents with new mechanisms of action that are apoptosis independent. 

**Figure 1:**
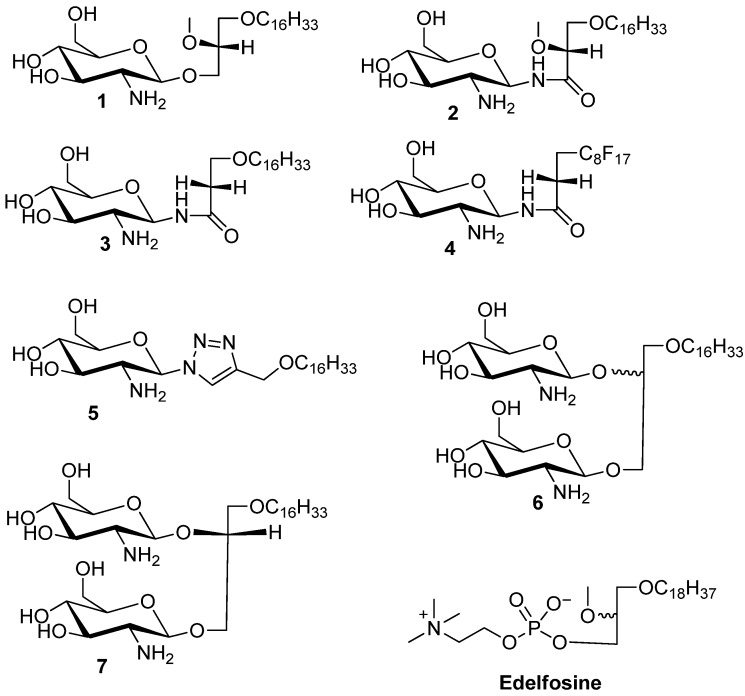
Structures of the synthesized glycolipids used in the study. Compound **1** is the reference GAEL while compounds **2**–**5** are glycolipid analogs differing in the nature of the glycosidic linkage and glycerol moiety. Compounds **6** and **7** are glycolipid analogs where the glycerolipid moiety contains two glycosidic linkages. Edelfosine is shown as a prototypic AEL analog.

Glycosylated antitumor ether lipids (GAELs), represent a subclass of antitumor ether lipids (AELs) in which the phosphocholine moiety of the prototypic compound, edelfosine is replaced by a sugar moiety [5]. The antitumor activity of these compounds has been well established [6]. Edelfosine is an investigational drug that has undergone phase II clinical trials. The best studied GAEL analog, glucosamine-based glycerolipid **1** (Figure 1) has potent antitumor effects against a range of cancer cell lines derived from breast, pancreas and prostate cancer with CC_50_ values in the range of 9–15 µM. This compound has been reported to kill cancer cells via an apoptosis independent pathway and is more active than the prototypic AELs, edelfosine, which kills cell via a apoptosis dependent pathway [6,7,8]. 

A major drawback for compound **1** is the lack of antitumor activity in *in vivo* studies using mice. A maximum tolerability dose could not be attained at concentrations of 500 mg/kg oral dosage or 4 mg/kg given intraperitoneally in Rag2M mice and there was no effect on the body weight of the animals [9]. Furthermore, at a dose of 100 mg/kg given orally and 4 mg/kg given intraperitoneally, it had no effect on the growth of JIMT-1 or MDA-MB231 xenografts growing in female Rag2M mice [9]. These observations are consistent with the hypothesis that the likely cause of the *in vivo* inactivity is due to cleavage of the β-*O*-glycosidic linkage by glucosidases. A *C*-glycosidic analogue of **1** has been synthesized to enhance the metabolic stability of the compound [10]. However the synthesis is very involved and alternative facile approaches have to be considered to generate metabolically stable analogues. Based on the rationale that both glycosylamide- and glycosyltriazole linkages will be resistant towards glycosidic cleavage [11,12,13], we synthesized compounds **2**–**4** where the β-*O*-glycosidic linkage was replaced by a glycosylamide- or a triazole-linkage as in compound **5**. In addition, we synthesized compounds **6** and **7** to study the effects of: (a) diglycosylation and (b) stereochemistry of the glycerol moiety on antitumor activity in a cell based assay. The rationale behind the synthesis of compounds **6** and **7** is to test our hypothesis that an additional glucosamine moiety on the molecule will increase the anticancer activity because glucosamine is cytotoxic against human epidermoid carcinoma cells in tissue culture [14] and YD-8 human oral cancer cells [15]. Here we report on the *in vitro* antitumor activity of compounds **2**–**7**. 

## 2. Results and Discussion

### 2.1. Chemistry

In order to prepare easily accessible analogs with improved metabolic stability towards glycosidases we initially were interested in analogs that contain a glycosylamide linkage. Glycosylamides **2**–**4** were synthesized to evaluate the effect of amide linkage and nature of the lipid moiety on the antitumor properties. Compound **3** which lacks the methoxy substituent was prepared to explore how the methoxy group affects the antitumor properties. Compound **4** that contains a lipophobic polyfluorinated lipid tail instead of a lipophilic carbon chain was prepared to explore how modifications in the lipid tail affect the antitumor activity. Compound **5** was synthesized to evaluate the effect of a triazole linkage at the anomeric position. Both linkages, the glycosylamide and glycosyltriazole linkages are expected to be metabolically stable to hydrolysis by glycosidases in *in vivo* studies [11,12,13]. In addition, the glycosyltriazole linkage will be inert towards peptidases and proteases which may provide additional benefits for future *in vivo* studies [16]. 

**Scheme 1:**
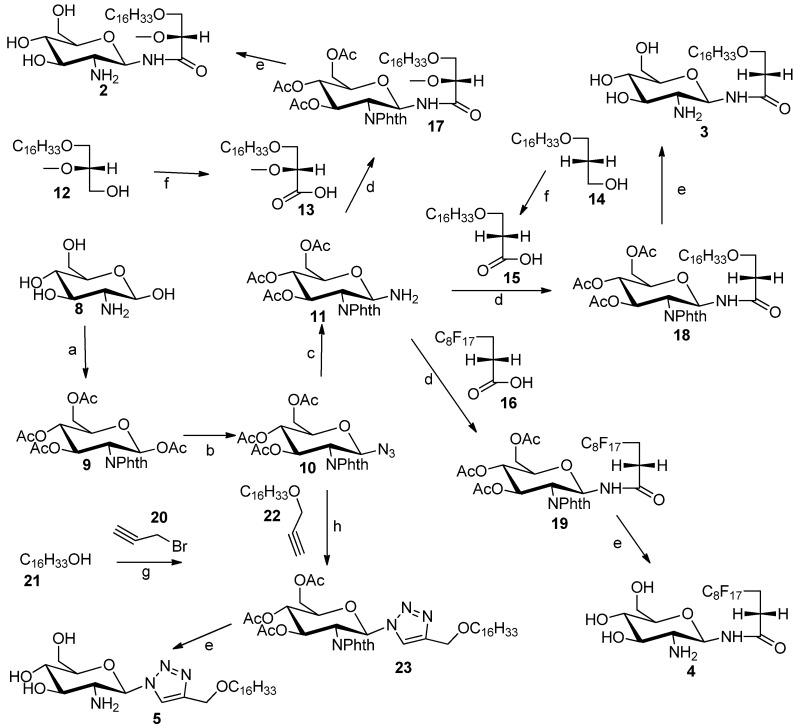
Synthesis of compounds **2**–**5**.

GAEL mimetics **2**–**4** were prepared by coupling of glycosylamine **11** to carboxylic acids **13**, **15** and **16** (Scheme 1). Glycosylamine **11** was synthesized in three steps from glucosamine hydrochloride (**8**) in 49% yield [17]. The amino substituent at C_2_ position of glucosamine hydrochloride group was protected with phthalic anhydride followed by protection of the hydroxyl groups as acetate esters by reaction with acetic anhydride in pyridine to afford compound **9**. The anomeric amino group in **11** was installed through conversion of **9** into the corresponding anomeric azide **10**. Originally, we planned to introduce the anomeric azido group by nucleophilic displacement of the α-anomeric chloride. However conversion of the anomeric acetate into the chloride by reaction with PCl_5_ did not afford the corresponding glucosylhalides [18]. However, the anomeric azide **10** was prepared by Fe(III) chloride- promoted reaction of **9** with trimethylsilyl azide to afford azide **10** in 75% yield. The anomeric azide was reduced to the amine by catalytic hydrogenation to provide glucosylamine **11** in 96% yield. Synthesis of the fatty acid compounds **13** and **15** was achieved by Jones oxidation of the corresponding commercially available alcohol **12** or known alcohol **14** [19]. The polyfluorinated fatty acid **16** was purchased from commercial source. Coupling of fatty acids **13**, **15 **and **16** to the glucosylamine **11** was achieved by using TBTU [20] as coupling agent to afford the protected glycolipids **17**, **18** and **19**, respectively. The acetate and phthalimido protecting groups were removed using an ethylenediamine-butanol mixture (1:1) at 90 °C for 3 h to afford desired target compounds **2**, **3** and **4** respectively. The triazole analog **5** was synthesized by Cu(I)-promoted click chemistry [21] using azide **10** and 3-hexadecyloxyprop-1-yne (**22**) to produce glycosyltriazole **23**. Deblocking using the same method as described above gave compound **5**.

*Reagents and conditions*: (a) (i) phthalic anhydride, NaOH, H_2_O, 16 h, rt; (ii) pyridine, DMAP, Ac_2_O, 16 h, rt; (b) TMSN_3_, FeCl_3_ as catalyst, DCM, rt; (c) Pd/C, MeOH, H_2_, 2 h, rt; (d) TBTU, DIPEA. DMF 3 h, rt; (e) ethylenediamine/butanol (1:1), 90 °C, 2 h; (f) Jones reagent, acetone, 0 °C, 20 min; (g) NaH, DMF, 80 °C; (h) CuI, acetic acid, DIPEA.

**Scheme 2:**
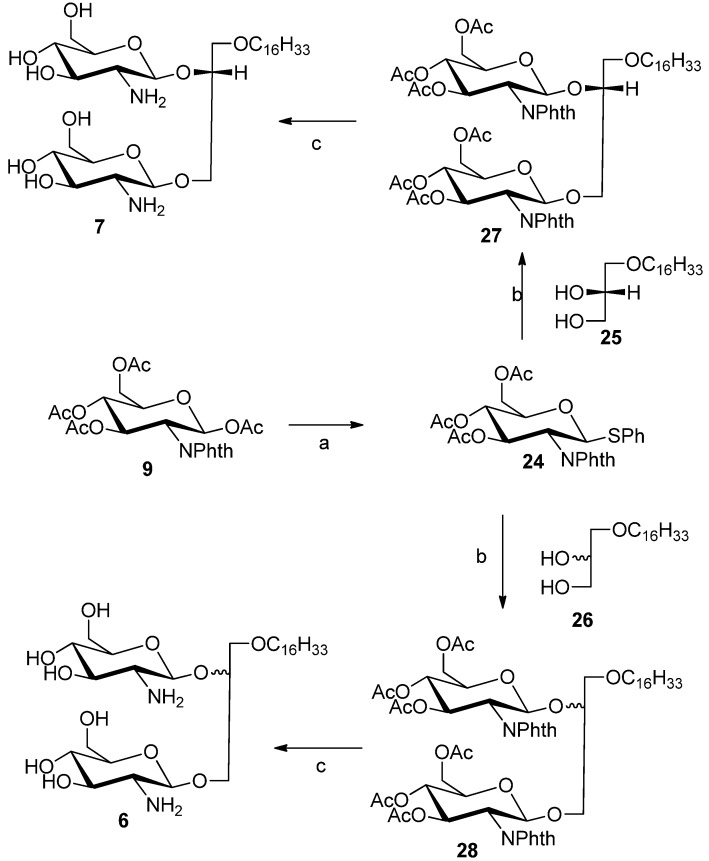
Synthesis of compounds **6** and **7**.

Compounds **6** and **7** were synthesized to study the effects of diglycosylation and stereochemistry of the glycerol moiety on the antitumor properties. Glycolipids **6** and **7** were prepared from known thioglycoside donor **24** [22] by glycosylation with commercially lipid alcohol **25** or racemic alcohol **26** using silver triflate as catalyst and *N*-iodosuccinimde as promoter to afford protected glycolipids **27** or **28**, respectively (Scheme 2). The acetate and phthalimido protective groups were removed using ethylenediamine:butanol mixture (1:1) at 90 °C for 3 h to provide compounds **6** and **7**, respectively. 

*Reactions and conditions*: (a) BF_3_·Et_2_O, DCM, reflux 16 h; (b) AgOTf, *N*-iodosuccinimide, DCM, rt, 5 h; (c) ethylenediamine/butanol (1:1), 90 °C, 4 h.

### 2.2. Cytotoxicity

**Figure 2:**
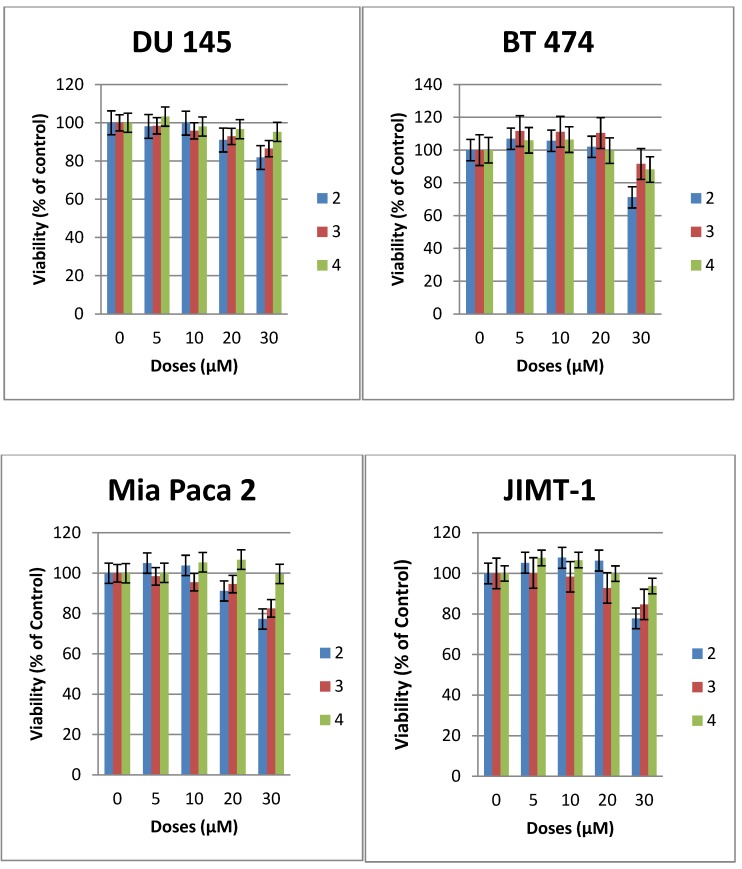
Effects of compounds **2**–**4** on the viability of epithelial cancer cell lines: breast (BT474, JIMT1), prostrate (DU 145), pancreas (MiaPaCa2). The results represent the means ± standard deviations of six independent determinations (compound **1** is not included in this chart because we have previously published the data [22]).

**Figure 3:**
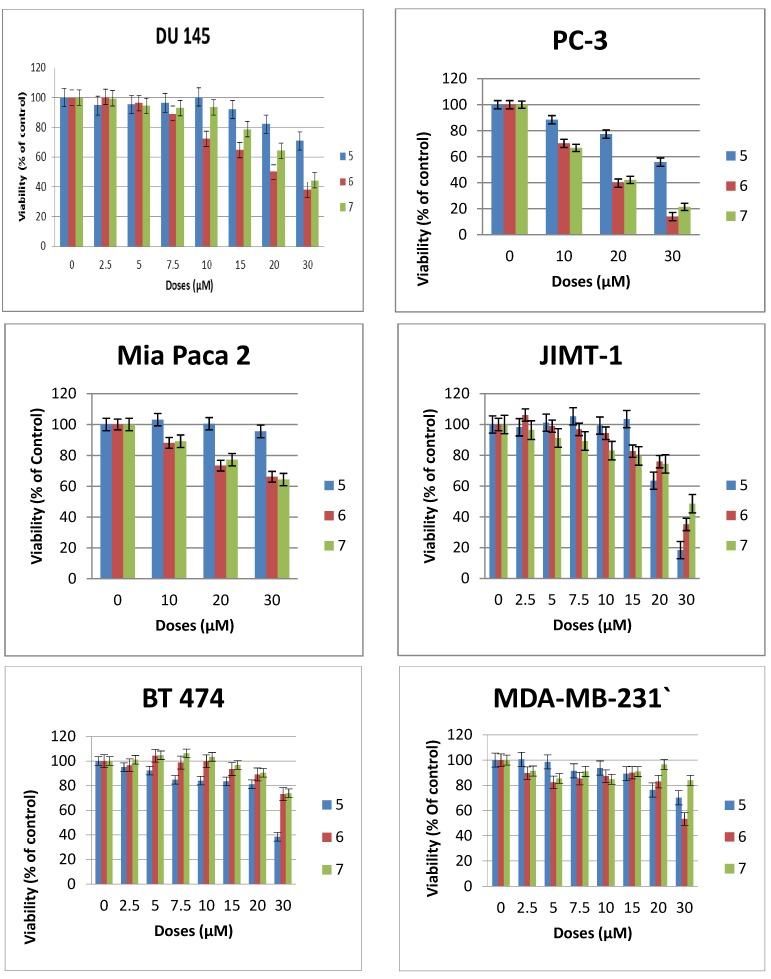
Effects of compounds **5**–**7** on the viability of epithelial cancer cell lines: breast (BT474, JIMT1, MDA-MB-231), prostrate (DU145, PC3), pancreas (MiaPaCa2). The results represent the means ± standard deviations of six independent determinations.

The cytotoxicity of compounds **1**–**7** was evaluated against a number of epithelial cancer cell lines using the MTS assay [22]. The cells were derived from cancers of the breast (JIMT-1, BT-474, MDA-MB-231), pancreas (MiaPaCa2), and prostrate (DU145, PC3). The cytotoxic effect of compounds **2**–**6** were compared with that of **1** the most studied glycosylated antitumor ether lipid [5,6,7,9] (see Table 1). Exponentially growing cells were treated with test compound and then incubated for 48 h. The result for compounds **2**–**4** are shown in Figure 2, while that of compounds **5**–**7** are shown in Figure 3. The CC_50_ values that lead to a reduction of cell viability by 50% relative to untreated control are reported in Table 1. At the highest concentration tested none of the *N*-linked glycolipids **2**–**4** was able to achieve 50% reduction in viability against all the four cell lines tested (see Figure 2). It is noteworthy that compound **2** with similar glycerolipid moiety as reference compound **1**, is consistently more active than compounds **3** and **4**, leading to a 19%–29% reduction in viability relative to untreated control at the highest dose tested (30 µM).

**Table 1:** Cytotoxicity of compounds **1**–**7** on a panel of human epithelial cancer cell lines: breast (BT474, JIMT1, MDA-MB-231), prostrate (DU145, PC3), pancreas (MiaPaCa2). The CC_50_ value is defined as the concentration required to decrease cell viability by 50% relative to the untreated control. Values were determined by MTS assay. The CC_50_ values were obtained by estimating the drug concentration at 50% viability on the y axis using line plots (graph not shown); NT = not tested. Please note that CC_50 _values for compound **1** were obtained from our previously published work [21].

Compd	CC_50_ (µM)					
	JIMT1	MiaPaCa2	DU145	PC3	BT474	MDA-MB-231
1	9	9	10	13.5	6.5	NT
2	>30	>30	>30	NT	>30	NT
3	>30	>30	>30	NT	>30	NT
4	>30	>30	>30	NT	>30	NT
5	23	>30	>30	>30	28	>30
6	27	>30	20	17.5	>30	>30
7	29	>30	25	17.5	>30	>30

At this dose and lower concentration, compound **4** with polyfluorinated lipid moiety is consistently the least active analogue among these *N*-linked (amide) glycolipids. This relative difference in activity is an indication that the nature of the lipids of this class of compounds plays an important role on their cytotoxicity against human cancer cell lines. The triazole-linked glycolipid compound **5** at concentration of 30 µM or below was unable to achieve CC_50_ values against prostrate and pancreas cell lines used in this experiment, indicating that both the nature of the glycosidic linkage and the glycero moiety influences the antitumor properties. It is noteworthy that compound **5** was able to achieve more than 80% reduction in viability of JIMT1, a trastuzumab resistant cell line [4] at the dose of 30 µM, (CC_50_: 23µM) and more than 60% reduction in viability of BT474, a trastuzumab sensitive cell lines (see Figure 3). *In vivo*, triazole-linked glycolipid **5** is expected to be metabolically stable relative to lead compound **1**, however the loss of activity does not make it a worthwhile compound for further development. 

The diglycosylated analogues, compounds **6** and **7 **with different stereochemistry at position 2 of the glycerol backbone have very similar activity but are significantly less active than GAEL-based reference compound **1** (Figure 2 and Table 1) indicating that additional glucosamine moieties on the glycerol backbone reduce the cytotoxic effect of the compounds. This reduction in activity may be due to increased polarity of the compound which in turn can affect the absorption of the drug across cell membrane, and subsequent decreased drug concentration in the cell. The fact that both compounds despite the difference in stereochemistry at the C-2 position of the glycerol backbone have similar activity especially in the more sensitive prostrate cell lines (Figure 2) is an indication that stereochemistry at this position has minimal to no effect on the anticancer activity. 

## 3. Experimental

### 3.1. General Methods

All fine chemicals such as glucosamine hydrochloride, phthalic anhydride, pyridine, 1-hexadecanol, 4-toluenesulfonyl chloride, 60% sodium hydride, dimethylformamide (DMF), 1,3-dihydroxypropane, sodium methoxide, concentrated sulfuric acid, palladium on carbon, trimethylsilyl azide, toluene, phosphorus pentachloride, boron trifluoride, *N*,*N*-diisopropyl-ethylamine, ethylenediamine and iron (III) chloride. All solvents such as dichloromethane (DCM), hexane, acetone, methanol, ethyl acetate, butanol were purchased from Sigma-Aldrich (Oakville, ON, Canada). Carboxylic acid **16** (2*H*,2*H*,3*H*,3*H*-perfluoroundecanoic acid) were purchased from Fluorous Technologies now available through Sigma-Aldrich. 1-*O*-hexadecyl-2-*O*-methyl-*sn*-glycerol was purchased from Chem-Implex (Wood Dale, IL, USA). 5 ¾ and 9 inch pipets used in lab were obtained from Fisher Scientific (Ottawa, ON, Canada). TLC plates (CCM Gel silica 60 F254) were visualized by ultraviolet light or by charring (9:1 methanol and sulfuric acid). All chromatography solvents were prepared by mixing hexane and ethyl acetate in various ratios based on the polarity of the compound. Column chromatography was performed by using silica gel p60 (40–63 µm) or reverse-phase C18 silica gel. Solutions were concentrated on reduced pressure rotary evaporator (IKA RV 10 Basic, that was connected to building vacuum and high vacuum pump (Welch 8907). ^1^H-NMR and ^13^C-NMR spectra were recorded on a 300 MHz (Bruker AMX-300 spectrometer). Low-resolution mass spectrometry (MS) data were obtained on a Varian 500-MS IT Mass spectrometer using electrospray ionization (ESI).

### 3.2. General Procedure for the Synthesis of N-Linked Compounds **2**–**5**

*1,3,4,6-Tetra-O-acetyl-2-deoxy-2-N-phthalimido-β-d-glucopyranoside* (**9**). Glucosamine hydro-chloride **8** (3.016 g, 14 mmol) and NaOH (28 mmol) were dissolved in water (50 mL). The resulting mixture was stirred at room temperature for 30 min. Phthalic anhydride (2.34 g, 0.0157 mol) was added to the solution. The mixture was stirred vigorously at room temperature for 16 h. The mixture was concentrated and dried using rotary evaporator. The residue was dissolved in pyridine (30 mL), and then Ac_2_O (19.8 mL) was added to the solution. The resulting solution was allowed to stir vigorously overnight. The reaction was checked by the TLC. Methanol (6 mL) was used to quench the excess of Ac_2_O, and then excess pyridine was removed under high vacuum. The remaining solid was dissolved in CH_2_Cl_2_ (40 mL), and then the solution was washed with 10% HCl (40 mL×1), Saturated NaHCO_3_ solution (40 mL×3), H_2_O (40 mL×1) and brine (40 mL×1) and dried over anhydrous MgSO_4_. The final solution was concentrated under reduced pressure, and the obtained product **8** (3.3g, 49.4%) was dried overnight. NMR data were consistent with data in the literature [23]. 

*3,4,6-tri-O-Acetyl-2-deoxy-2-N-phthalimido-β-d-glucopyranosyl azide* (**10**). Compound **9** (1.8 g, 8 mmol) and trimethylsilyl azide (1.7 g, 148 mmol) were both dissolved in CH_2_Cl_2 _(20 mL) in a 100 mL-round bottom flask with vigorous stirring. Then FeCl_3_ (1.77 g) was added to the reacting mixture. The reaction was allowed to stir for 24 h and then progress was checked by TLC. The dispersion solution was made by mixing hexane and ethyl acetate in 1:1 ratio. The solution was concentrated under reduced pressure by rotary evaporator. The product **10** was isolated and purified by column chromatography (1:2 ethyl acetate/hexane). The obtained product **10** (1.3 g, 76.53%) was a light yellow solid. NMR data were consistent with previously published data [16].

*3,4,6-tri-O-Acetyl-1-amino-2-deoxy-2-N-phthalimido-β-d-glucopyranose* (**11**). Compound **10** (0.11 g, 0.24 mmol) was dissolved in methanol (2 mL) in a 25 mL-round bottom flask with vigorous stirring, and then Pd/C (0.29 g) was added. After that, round bottom flask was connected to a hydrogen balloon. The reaction was allowed to take place for half hour, and checked by TLC. The reaction was stopped when all starting material has disappeared, and the solution was concentrated to provide compound **11** (100 mg, 96%). This compound was not characterised by ^1^H-NMR, because it is chemically unstable. Therefore it was directly used for the next step, *i.e.*, coupling of carboxylic acids using TBTU as coupling reagent.

*3-(Hexadecyloxy)-2-methoxypropanoic acid* (**13**). A 2.7 M solution of Jones reagent (0.5 mL) was added dropwise into a stirred solution of commercially available 3-*O*-hexadecyloxy-2-*O*-methyl-sn-glycerol **12** (23 mg) in acetone (5 mL) at 0 °C. The reaction was monitored by TLC and was completed after 1 h. Isopropyl alcohol was added dropwise until a green color remained, to remove excess of CrO_3_ totally. The organic solvent was removed under reduced pressure. The solid was dissolved in water, and then 3-(hexadecyloxy)-2-methoxypropanoic acid was extracted using ethyl acetate. The combined organic layers were washed with brine, dried using MgSO_4_ and concentrated under vacuum. The 3-(hexadecyloxy)-2-methoxypropanoic acid (**13**) was purified by silica gel column flash chromatography (4:1 hexane/ethyl acetate) to yield white solid (16 mg, 70%). ^1^H-NMR (CDCl_3_): δ = 4.00–3.75 (m, 3H), 3.55 (s, 3H, –OCH_3_), 2.66 (t, *J* = 6.4 Hz, 2H, –O**CH_2_**–CH_2_–(CH_2_)_13_), 1.59 (m, 2H, –CH_2_–(CH_2_)_13_), 1.23–1.34 (brs, 26H, –(CH_2_)_13_), 0.90 (t, *J* = 6.9 Hz, 3H, –CH_3_). ESMS: calcd for C_20_H_40_O_4_Na^+^ *m/z* 367.3; found: *m/z* [M+Na]^+^ 367.3.

*3-(Hexadecyloxy)-propanoic acid* (**15**). A 2.7 M solution of Jones reagent (2.0 mL) was added dropwise into a stirred solution of compound **14** (0.124 g, 0.4 mmol) in acetone (20 mL) at 0 °C. The reaction was monitored by TLC and was completed after 1 h. Isopropyl alcohol was added dropwise until a stable green color appeared, to remove the excess of CrO_3_. The organic solvent was removed under reduced pressure. The solid was dissolved in water, and then compound **15** was extracted using ethyl acetate. The combined organic layers were washed by brine, dried using MgSO_4_ and concentrated under reduced pressure. The compound **15** was purified by silica gel column flash chromatography (4:1 hexane/ethyl acetate) to yield white solid (63 mg, 50.1%). ^1^H-NMR (CDCl_3_): δ = 3.72 (t, *J* = 6.3 Hz, 2H, –**CH_2_**–COOH), 3.48 (t, *J* = 6.7 Hz, 2H, –O**CH_2_**–CH_2_–COOH), 2.65 (t, *J* = 6.3 Hz, 2H, –O**CH_2_**–CH_2_–(CH_2_)_13_), 1.59 (m, 2H, –**CH_2_**–(CH_2_)_13_), 1.23–1.34 (brs, 26H, –(CH2)_13_ 0.90 (t, *J* = 6.9 Hz, 3H, CH_3_). ESMS: calcd for C_19_H_38_O_3_Na^+^ *m/z* 337.3; found: *m/z* [M+Na]^+^ 337.4

*3-Hexadecyloxy-2-methoxyl-1-N-(3,4,6-tri-O-acetyl-2-deoxy-2-N-phthalimido-β-d-glucopyranosyl)-propanamide* (**17**). Glucosylamine **11** (110 mg, 0.25 mmol), carboxylic acid **13 **(110 mg, 0.32 mmol), DIPEA (0.40 mL) and TBTU (0.245 g) were dissolved in DMF (10 mL) in a round bottom flask with vigorous stirring overnight and was monitored by TLC. At the completion of the reaction DMF was removed under reduced pressure to obtain a solid residue. The solid residue was dissolved in water, and the product was extracted by ethyl acetate. The combined organic layers were concentrated under reduced pressure to yield a light yellow compound **17** (130 mg, 68%). ^1^H-NMR (CDCl_3_): δ = 8.84 (s, br. 1H), 7.93–7.67 (m, 4H, phthalimido), 7.11(d, *J* = 9.0 Hz, 1H, H–1), 6.14–5.96 (m, 2H, H–2, H–3), 5.19 (dd, *J_1_ = J_2_* = 12 Hz, 1H, H–4), 4.44–3.54 (m, 8H), 3.26 (s, 3H, –OCH_3_), 2.12(s, 3H, acetate–CH_3_), 2.06 (s, 3H, acetate–CH_3_), 1.89 (s, 3H, acetate–CH_3_), 1.53 (m, 2H–**CH_2_**–(CH_2_)_13_), 1.39–1.15 (brs, 26H, (CH_2_)_13_), 0.90 (t, *J* = 6.0 Hz, 3H, CH_3_). ES-MS: calcd for C_40_H_60_N_2_O_12_Na^+^ *m/z* 783.4: found: *m/z* [M+Na]^+^ 783.6.

*3-Hexadecyloxy-1-N-(3,4,6-tri-O-acetyl-2-deoxy-2-N-phthalimido-β-d-glucopyranosyl)-propanamide* (**18**). Glucosylamine **11** (100 mg, 0.23 mmol) and carboxylic acid **15** (43.7 mg, 0.15 mmol) and DIPEA (0.04 g) and TBTU (0.04 g) and DMF (4 mL) were added into a 25-mL round bottom flask with vigorous stirring. The reaction allowed to stir for overnight and was monitored by TLC. DMF was removed under reduced pressure to obtain solid. The solid was dissolved in water, and the product was extracted by ethyl acetate. The combined organic layers were concentrated by reduced pressure rotary evaporator to yield a light yellow compound **18** (50 mg, 45.5%). ^1^H-NMR (CDCl_3_): δ = 8.84 (s, br. 1H), 7.89–7.76 (m, 4H, phthalimido), 7.03 (d, *J =* 9.0 Hz, 1H, H–1), 6.08 (dd, *J_1_ = J_2_* = 9.8 Hz, 1H, H-3), 6.02 (dd, *J_1_ = J_2_ =* 9.6 Hz, 1H, H–2), 5.18 (dd, *J_1_ = J_2_* = 9.8 Hz, 1H, H–4), 4.36 (m, 1H, H–5), 4.28 (dd, *J_1_ = J_2_ =* 10.2 Hz, 2H, ), 4.09 (dd, *J_1_ = J_2_ = 10.0* Hz, 2H, H–6), 3.51 (m, 1H), 3.34 (m, 2H), 2.26 (m, 2H,), 2.12 (s, 3H, acetate–CH_3_), 2.05 (s, 3H, acetate–CH_3_), 1.88 (s, 3H, acetate–CH_3_), 1.53 (m, 2H, CH_2_–(CH_2_)_13_), 1.31–1.28 (brs, 26H, –(CH_2_)_13_), 0.90 (t, *J* = 6.9 Hz, 3H, –CH_3_). ES-MS: calcd for C_39_H_58_N_2_O_11_Na^+^ *m/z* 753.4: found: *m/z* [M+Na]^+^ 753.8.

*Heptadecylfluoro-1-N-(3,4,6-tri-O-acetyl-2-deoxy-2-N-phthalimido-β-d-glucopyranosyl)-undecanamide* (**19**). Glucosylamine **11** (110 mg, 0.25 mmol), fluorinated carboxylic acid (250 mg, 0.51 mmol), DIPEA (0.04 g), TBTU (0.245 g) and DMF (10 mL) were added into a 25-mL round bottom flask with vigorous stirring. The reaction allowed to stir for overnight and was monitored by TLC. DMF was removed under reduced pressure to obtain solid. The solid was dissolved in water, and the product was extracted by ethyl acetate. The combined organic layers were concentrated by reduced pressure rotary evaporator to yield a light yellow compound **19** (105 mg, 45%). ^1^H-NMR (CDCl_3_): δ = 8.93 (s, br. 1H), 7.93–7.69 (m, 4H, phthalimido), 7.05 (d, *J* = 9.0 Hz, 1H, H–1), 6.15–5.99 (m, 2H, H–2, H–3), 5.17 (dd, *J_1_ = J_2_* = 9.0 Hz, 1H, H–4), 4.45–3.98 (m, 5H), 2.38 (m, 2H), 2.13 (s, 3H, acetate–CH_3_), 2.06 (s, 3H, acetate–CH_3_), 1.89 (s, 3H, acetate–CH_3_). ES-MS: calcd for C_31_H_25_F_17_N_2_O_10_Na^+^ *m/z* 931.1: found: *m/z* [M+Na]^+^ 931.5.

*3-Hexadecyloxy-2-methoxy-1-N-(-2-deoxy-2-amino-β-d-glucopyranosyl)-propanamide* (**2**). Compound **17** (130 mg, 0.17 mmol) was dissolved in mixture of butanol (2 mL) and ethylenediamine (2 mL). The resulting mixture was heated to 90 °C and stirred for 3 h. The reaction was monitored by TLC plate. The solution was concentrated to dryness. The resulting reside was purified by flash chromatography (reverse-phase C18 silica gel). The collected product was 20 mg (yield 23%). The compound was acidified with TFA to convert it into the TFA salt. ^1^H-NMR (CD_3_OD): δ = 5.14 (d, *J* = 9.6 Hz, 1H, H–1), 3.87–3.52 (m, 6H), 3.50–3.24 (m, 7H, H–3), 3.08 (dd, *J =* 9.6 Hz, 10 Hz, 1H, H–2), 1.56–1.36 (m, 2H, CH_2_–(CH_2_)_13_), 1.25–1.13 (brs, 26H, –(CH_2_)_13_), 0.80 (t, *J* = 6.9 Hz, 3H, –CH_3_). ^13^C-NMR (75 MHz, CD_3_OD) characteristic data: 174.03 (C–1), 82.50, 80.41, 78.00, 74.87, 72.88, 71.49, 71.25, 62.11, 58.94 56.46, + multiple methylene carbons, 14.40 (CH_3_). ES-HRMS: calcd for C_26_H_52_N_2_O_7_Na^+^ *m/z* 527.3667, found: *m/z* [M+Na]^+^ 527.3677.

*3-Hexadecyloxy-1-N-(-2-deoxy-2-amino-β-d-glucopyranosyl)-propanamide* (**3**). Compound **8** (50 mg, 0.07 mmol) was dissolved in mixture of butanol (2 mL) and ethylenediamine (2 mL). The resulting mixture was heated to 90 °C and stirred for 3 h. The reaction was monitored by TLC. The solution was concentrated to dryness. The resulting reside was purified by flash chromatography (reverse-phase C18 silica gel). The collected product was 18 mg (yield 54%). The compound **3** was acidified with TFA to enhance solubility in methanol. ^1^H-NMR (CD_3_OD): δ = 5.14 (d, *J* = 10 Hz, 1H, H–1), 3.74–3.56 (m, 4H), 3.48–3.42 (m, 1H), 3.40–3.32 (m, 2H), 3.30–3.25 (m, 2H), 2.89 (dd, *J_1_ = J_2_ =* 10.0 Hz, 1H, H–2), 2.44 (t, *J =* 6 Hz, 2H,), 1.51–1.42 (m, 2H, –CH_2_–(CH_2_)_13_), 1.25–1.18 (brs, 26 H, –(CH_2_)_13_), 0.81 (t, *J* = 7 Hz, 3H, –CH_3_). ^13^C-NMR (CD_3_OD) characteristic data: 174.50 (amide), 80.30 77.83, 74.87, 72.43, 71.43, 67.11, 62.18, 56.90, 37.54, (multiple methylene carbons), 14.43 (CH_3_). ES-HRMS: calcd for C_25_H_50_N_2_O_6_Na^+^ *m/z* 497.3567: found: *m/z* [M+Na]^+^ 497.3554.

*Heptadecylfluoro-1-N-(-2-deoxy-2-amino-β-d-glucopyranosyl)-dodecanamide* (**4**). Compound **19** (105 mg, 0.11 mmol) was dissolved in mixture of butanol (2 mL) and ethylenediamine (2 mL). The resulting mixture was heated to 90 °C and stirred for 3 h. The reaction was monitored by TLC plate. The solution was concentrated to dryness. The resulting residue was purified by flash chromatography (reverse-phase C18 silica gel) to yield product **4** (50 mg, 64% yield). The compound **3** was acidified with TFA to enhance solubility in methanol. ^1^H-NMR (CD_3_OD): δ = 5.16 (d, *J* = 9.9 Hz, 1H, H–1), 3.80–3.73 (m, 1H, H–6a), 3.65–3.55 (m, 1H, H–6b), 3.52–3.43 (m, 1H, H–5), 3.34–3.17 (m, 4H), 2.91 (dd, *J*_1_ = 9.9 Hz, *J*_2_ = 9.9 Hz, 1H, H–2), 2.59–2.33 (m, 2H). ^13^C-NMR (CD_3_OD) for sugar carbons 173.32 (amide), 80.33, 77.96, 74.88, 71.45, 62.21, 56.87. ES-HRMS: calcd for C_17_H_17_F_17_N_2_O_5_Na^+^ *m/z* 675.0758: found: *m/z* [M+Na]^+^ 675.0786.

*3-Hexadecyloxyl-propy-1-ne* (**22**). Compound **20** (8.25 mmol, 2.0 g) and **21** (9.9 mmol, 0.884 mL) were dissolved in dry DMF (20 mL) under nitrogen atmosphere, then NaH (9.9 mmol, 237 mg) was added and the reaction mixture was heated to 90 °C overnight. The reaction was stopped by addition of water (8 mL). The mixture was concentrated under high vacuum and the residue was purified by flash chromatography using hexane/ethyl acetate mixture (9.5:0.5) to give compound **22** as a white sticky solid yield 60%. The NMR data correspond to the previously reported data [24].

*4-(Hexadecyloxy)methyl)-1-(3,4,6-tri-O-acetyl-2'-deoxy-2'-N-phthalimido-β-d-glucopyranosyl)-1,2,3-triazole* (**23**). Compound **10** (0.09 mmol, 40 mg) and compound **22** (0.1 mmol, 29 mg) were dissolved in DCM (5 mL), copper-I-iodide (0.009 mmol, 1.65 mg), acetic acid (0.17 mmol, 12.88 mg), and diisopropylethylamine (0.17 mmol, 22.48 mg) were added at the same time and the reaction mixture were left stirring for 6 h. At the end of the reaction (TLC monitoring), the reaction mixture was concentrated under vacuum and purified by flash chromatography using a hexane/ethyl acetate mixture (6:4) to give compound **23** as a white solid. Yield 58 mg (90%). ^1^H-NMR (CDCl_3_): δ = 7.81–7.71 (m, 5H, phthalimido and triazole), 6.79 (d, *J* = 9.9 Hz, 1H, H–1), 6.03 (dd, *J_1_* = 10.2 Hz, *J_2_* *=* 9.6 Hz, 1H, H–3), 5.34 (dd, *J_1_* = *J_2_* *=* 9.6Hz, 1H, H–4), 4.85 (dd, *J_1_* = 9.9 Hz, *J_2_* = 10.2 Hz, 1H, H–2), 4.55 (s, 2H, –CH_2_–O–CH_2_), 4.37 (m, 1H, H–5), 4.26–4.05 (m, 2H, H–6), 3.42 (t, *J* = 6.7 Hz, 2H), 2.14 (s, 3H, acetate–CH_3_), 2.05 (s, 3H, acetate–CH_3_), 2.01 (s, 3H, acetate–CH_3_), 1.45–1.55 (m, 2H, CH_2_–(CH_2_)_13_), 1.25 (brs, 26 H, –(CH_2_)_13_), 0.88 (t, *J* = 6.6 Hz, 3H, –(CH_2_)_13_–CH_3_). ^13^C-NMR (CDCl_3_) δ 171.18, 170.59, 169.91, 169.43, 146.16, 134.56, 123.90, 120.75, 83.03, 77.50, 77.28, 77.08, 76.65, 75.05, 73.58, 70.96, 70.47, 68.24, 64.05, 61.69, 60.40, 54.03, 31.92, 29.69, 29.65, 29.59, 29.49, 29.35, 26.07, 22.68, 21.03, 20.71, 20.66, 20.58, 20.34, 14.19, 14.11. ESMS: calcd for C_39_H_56_N_4_O_10_Na ^+^ *m/z*: 763.4, found *m/z* [M+Na]^+^ 763.7.

*4-(Hexadecyloxy)methyl)-1-(2'-deoxy-2'-amino-β-d-glucopyranosyl)-1,2,3-triazole* (**5**). Compound **23** (58 mg) was dissolved in mixture of butanol (2 mL) and ethylenediamine (2 mL). The resulting mixture was heated to 90 °C and stirred for 3 h. The reaction was monitored by TLC. The solution was concentrated to dryness. The resulting residue was purified by flash gradient chromatography (reverse-phase C18 silica gel, 100 water to 100 methanol) to give compound **5** as a white solid in a yield 22 mg (60%). ^1^H-NMR (CD_3_OD) δ = 8.21 (s, 1H, triazole H), 5.60 (d, *J* = 9.2 Hz, 1H, H–1), 3.91 (dd, *J* = 12.2, 12.1 Hz, 1H H–3), 3.74 (dd, *J* = 12.2, 5.0 Hz, 1H, H–4), 3.67–3.25 (m, 8H), 1.69–1.53 (m, 2H, –CH_2_–(CH_2_)_13_), 1.32 (brs, 26H, –(CH_2_)_13_), 0.93 (t, *J* = 6.9 Hz, 3H, –CH_3_). ^ 13^C-NMR (CD_3_OD) δ = 124.58 (triazole–CH), 81.27 (–C1), 71.85, 64.63, 62.43, 49.89, 49.60, 49.32, 49.04, 48.75, 48.47, 48.19, 33.09, 30.79, 30.75, 30.62, 30.48, 27.24, 23.75, 14.47. ES-HRMS: calcd for C_25_H_48_N_4_O_5_Na^+^ *m/z* 507.3517: found: *m/z* [M+Na]^+^ 507.3532.

### 3.3. General Procedure for the Synthesis of Diglycosylated Compounds **6** and **7**

Compounds **6** and **7** were synthesized by diglycosylation of commercially available lipids **25** and racemic alcohol **26**, respectively. The previously reported thioglycoside donor **24** [19] was synthesized from compound **9** and thiophenol using BF_3_^.^Et_2_O as promoter in DCM at 60 °C for 16 h. The glycosylation reaction was carried out using silver triflate and *N*-Iodosuccinimide in anhydrous DCM under argon atmosphere for 5 h. The reaction was stopped by addition of saturated sodium thiosulphate solution followed by washing with saturated NaHCO_3 _solution (×3). The organic layer was concentrated under vacuum to give a brownish residue which was purified with flash chromatography using hexane/ethyl acetate mixture (6:4) to give protected diglycosylated glycolipids **27** and **28 **as a white foam (yield 45%). Compounds **27** and **28** were subsequently deprotected using a 1:1 mixture of ethylenediamine and butanol for 4 h, followed by removal of solvent under vacuum and purified with Ethyl acetate/methanol mixture (7:3) to give compounds **6** and **7**, respectively (yield 70%).

*3-Hexadecyloxy-1,2R-di(3,4,6-tri-O-acetyl-2'-deoxy-2'-N-phthalimido-β-d-glucopyranosyl)-glycerol* (**27**). ^1^H-NMR (CDCl_3_) δ = 7.93–7.71 (m, 8H, phthalimido), 5.75–5.62 (m, 2H), 5.53 (d, *J =* 8.5 Hz, 1H), 5.17 (d, *J* = 8.7 Hz, 1H), 5.14–5.00 (m, 2H), 4.41–4.38 (m, 2H), 4.26–4.09 (m, 3H), 3.91–3.83 (m, 1H), 3.82–3.66 (m, 4H), 3.50–3.33 (m, 2H), 3.09–3.15 (m, 3H), 2.17 (s, 3H), 2.12 (s, 3H), 1.95 (d, *J* = 2.8 Hz, 6H, acetate–CH_3_), 1.91 (d, *J* = 2.8 Hz, 6H, acetate–CH_3_), 1.64 (m, 2H), 1.43–1.05 (brs, 26H, –(CH_3_)_13_), 0.88 (t, *J* = 6.6 Hz, 3H). ^13^C-NMR (CDCl_3_) δ = 171.22, 170.27, 169.74, 168.66, 165.86, 134.16, 123.51, 123.49, 98.76, 96.97, 77.56, 76.71, 71.64, 71.61, 70.66, 70.47, 69.89, 69.16, 68.91, 62.18, 54.61, 54.40, 37.02, 31.94, 29.72, 29.51, 29.38, 25.98, 22.70, 22.70, 20.80, 14.13. ESMS: Calcd for C_59_H_78_N_2_O_21_Na^+^ *m/z* 1173.5, found *m/z* [M+Na]^+^ 1173.6.

*3-Hexadecyloxy-1,2S/R-di(3,4,6-triacetyl-2'-deoxy-2'-N-phthalimido-β-d-glucopyranosyl)-glycerol* (**28**). The NMR data of compound **28** shown here are for the 2*R* and 2*S* 1:1 diastereomeric mixture and the four anomeric protons were identified and labeled accordingly. ^1^H-NMR (CDCl_3_) characteristic data: δ = 7.90–7.68 (m, 16H aromatic protons), 5.76–5.63 (m, 2H, H-3 (sugar 1), H-3 (sugar 2), 5.53 (d, *J =* 8.5 Hz, 1H, H–1 (sugar 1-2*R*-isomer), 5.39 (d, *J* = 8.5 Hz, 1H, H–1 (sugar 1 2*S* isomer), 5.33 (d, *J* = 8.3 Hz, 1H, H–1 (sugar 2-2*S* isomer), 5.17 (d, *J* = 8.7 Hz, 1H, H–1 (sugar 2-2*R*-isomer), 1.43–1.05 (brs, 52H, –(CH_3_)_13_), 0.85 (t, *J* = 6.6 Hz, 6H). ^13^C-NMR (CDCl_3_) characteristic data: δ = 170.72, 170.05, 169.55, 167.80, 134.25, 131.47, 123.70, 123.41, 98.77, 98.36, 78.65, 71.76, 71.64, 71.37, 70.76, 70.71, 69.90, 69.17, 69.00, 68.91, 62.19, 62.02, 54.63, 54.42, 31.95, 29.63, 29.38, 28.94, 22.71, 20.80, 14.13. ESMS: Calcd for C_59_H_78_N_2_O_21_Na^+^ *m/z* 1173.5, found *m/z* [M+Na]^+^ 1173.5.

*3-Hexadecyloxy-1,2S/R-di(-2'-deoxy-2'-amino-β-d-glucopyranosyl)-glycerol* (**6**). ^1^H-NMR (CD_3_OD) characteristic data for the diastereomeric mixture: δ 4.83–4.73 (m, 2H, H–1 (sugar 1, 2*R*/2*S*), 4.71–4.57 (m, 2H, H–1 (sugar 2, 2*R*/2*S*), 1.59 (m, 4H, –CH_2_–(CH)_13_), 1.32 (brs, 52H, (CH_2_)_13_), 0.93 (2xt, 6H, −CH_3_). ^13^C-NMR (MeOD) δ 102.75, 102.01, 101.47, 100.89 (anomeric carbons) 78.82, 78.75, 78.69, 78.53, 78.12, 75.45, 75.16, 74.95, 73.18, 72.18, 72.14, 71.85, 69.97, 62.80, 62.71, 62.12, 61.93, 58.33, 58.16, 50.24, 49.67, 34.98, 34.86, 33.45, 31.16, 31.14, 31.06, 31.02, 30.84, 27.57, 24.65, 24.11, 19.13, 14.82. ES-HRMS: calcd for C_31_H_62_N_2_O_11_Na^+^ *m/z* 661.4246,found: *m/z* [M+Na]^+^ 661.4241.

*3-Hexadecyloxy-1,2R-di(-2'-deoxy-2'-amino-β-d-glucopyranosyl)-glycerol* (**7**). ^1^H-NMR (methanol-*d*_4_) δ = 4.78 (dd, *J* = 8.3, 7.9 Hz, 1H, H–1a), 4.68 (dd, *J* = 8.3 Hz, 7.8 Hz, 1H, H–1b) 4.27–4.19 (m, 1H), 4.09–3.90 (m, 4H), 3.85–3.62 (m, 4H), 3.62–3.45 (m, 4H), 3.45–3.25 (m, 4H), 2.98–2.79 (m, 2H, H–2a, H–2b), 1.66–1.53 (m, 2H, –CH_2_–CH_2_–(CH_2_)_13_), 1.32 (brs, 26H, –(CH_2_)_13_–), 0.93 (t, *J* = 6.8 Hz, 3H, –CH_3_). ^13^C-NMR (CD_3_OD) δ = 101.92, 100.38, 78.17, 77.74, 74.40, 71.83, 71.69, 71.35, 71.24, 69.52, 67.17, 62.37, 57.95, 57.71, 35.99, 33.08, 30.80, 27.19, 23.74, 14.44. ES-HRMS: calcd for C_31_H_62_N_2_O_11_Na^+^ *m/z* 661.4246, found: *m/z* [M+Na]^+^ 661.4222.

### 3.4. Biological Activity

#### 3.4.1. Cell Culture

Breast (BT474, MDA-MB-231), prostrate (DU 145, PC3), pancreas (MiaPaCa2) cell lines were grown from frozen stocks of cells that were originally obtained from ATCC (Manassas, VA, USA). JIMT-1 breast cancer cells were originally obtained from DSMZ (Braunschweig, Germany). JIMT1, DU145 cells were grown in Dulbecco’s modified Eagle’s medium (DMEM), PC3 cells were grown in F12K medium. The cells were grown in media supplemented with 10% fetal bovine serum (FBS), penicillin (100 U/mL) and streptomycin (0.1 mg/mL)

#### 3.4.2. Cytotoxicity Assay

The cytotoxicity assay was carried out using a previously reported method [22]. Cell viability was determined with the cell Titre 96 AQueous One Solution (MTS assay, Promega, Madison, WI, USA. Equal numbers of cancer cells (7500–9500) in media (100 µL) were dispersed into 96-well plates. As blanks, media without cells (100 µL) were also placed in some wells and treated similarly to the cell containing wells. After an incubation period of 24 h, a solution of test compound ((100 µL) in medium at twice the desired concentration was added to each well. The treated cells were incubated for a further 48 h, after which time methanethiosulfonate (MTS) reagent (20%^V^/_V_) was added to each well. The plates were incubated for 1–4 h on a Nutating mixer in a 5% CO_2_ incubator, and then the optical density (OD) was read at 490 nm by using a SpectraMax M2 plate reader (Molecular Devices Corp., Sunnyvale, CA, USA). The blank values were substracted from each value, and the viability values of the treated samples relative to controls with vehicle were calculated. The values for the plots are the means ± standard deviation of six different wells. 

## 4. Conclusions

Six novel cationic GAEL analogs were synthesized to explore novel SAR in this class of compounds. We were especially interested to explore how changes in the nature of the anomeric linkage, the nature of the hydrophobic lipid tail and the stereochemistry of the glycerol moiety affects the cytotoxic properties against breast, pancreas and prostate cancer cell lines. During our study, we discovered that replacement of the *O*-glycosidic linkage of lead compound **1** with an *N*-glycosidic linkage or a triazole linkage resulted in significant loss of anticancer activity against all six cancer cell lines tested. Moreover, replacement of the hydrophobic lipid tail by a fluorinated tail as well as replacement of the methoxy substituent by a second glucosamine moiety resulted in decreased cytotoxic activity.
